# Increased mRNA expression levels of *ERCC1*, *OGG1 *and *RAI *in colorectal adenomas and carcinomas

**DOI:** 10.1186/1471-2407-6-208

**Published:** 2006-08-16

**Authors:** Mona Sæbø, Camilla Furu Skjelbred, Bjørn Andersen Nexø, Håkan Wallin, Inger-Lise Hansteen, Ulla Vogel, Elin H Kure

**Affiliations:** 1 Telemark University College, Faculty of Arts and Sciences, Department of Environmental and Health Studies, Hallvard Eikas plass, N-3800 Bø i Telemark, Norway; 2 Department of Laboratory Medicine, Section of Medical Genetics, Telemark Hospital, N-3710 Skien, Norway; 3 Institute of Human Genetics, University of Aarhus, Aarhus, Denmark; 4 National Institute of Occupational Health, Copenhagen, Denmark; 5 Department of Pathology, Ullevaal University Hospital, Oslo, Norway

## Abstract

**Background:**

The majority of colorectal cancer (CRC) cases develop through the adenoma-carcinoma pathway. If an increase in DNA repair expression is detected in both early adenomas and carcinomas it may indicate that low repair capacity in the normal mucosa is a risk factor for adenoma formation.

**Methods:**

We have examined mRNA expression of two DNA repair genes, *ERCC1 *and *OGG1 *as well as the putative apoptosis controlling gene *RAI*, in normal tissues and lesions from 36 cases with adenomas (mild/moderat n = 21 and severe n = 15, dysplasia) and 9 with carcinomas.

**Results:**

Comparing expression levels of *ERCC1, OGG1 *and *RAI *between normal tissue and all lesions combined yielded higher expression levels in lesions, 3.3-fold higher (P = 0.005), 5.6-fold higher(P < 3·10^-5^) and 7.7-fold higher (P = 0.0005), respectively. The levels of *ERCC1, OGG1 *and *RAI *expressions when comparing lesions, did not differ between adenomas and CRC cases, P = 0.836, P = 0.341 and P = 0.909, respectively. When comparing expression levels in normal tissue, the levels for *OGG1 *and *RAI *from CRC cases were significantly lower compared to the cases with adenomas, P = 0.012 and P = 0.011, respectively.

**Conclusion:**

Our results suggest that increased expression of defense genes is an early event in the progression of colorectal adenomas to carcinomas.

## Background

Most colorectal cancers (CRC) develop through multiple mutations in the normal colonic mucosa, and evolve through the adenoma-carcinoma sequence [1,2]. Various endogenous and exogenous agents from environmental exposures are constantly damaging DNA, and in combination with low DNA repair capacity this have been interpreted as increasing the likelihood of cancer development [3-7].

The range of different DNA lesions is broad and there are different repair pathways depending on the nature of the damage. Nucleotide excision repair (NER) will repair bulky adducts [8] while base excision repair (BER) is responsible for the repair of base damage and single strand breaks [9]. Only a few studies have to our knowledge studied DNA repair expression of nucleotide excision repair (NER) and base excision repair (BER) genes in CRC. Even less is known about the level and significance of DNA repair expression during the formation of adenomas and the transition period in the adenoma-carcinoma sequence. Previous studies have indicated that the NER gene excision repair cross-complementation group1 (ERCC1) and BER gene 8-oxoguanine glycosylase 1 (*OGG1*) may be inducible [10] and they have also been shown to correlate with enhanced DNA repair activities [11-15]. DNA repair mechanisms are complex and involve several genes. *OGG1 *and *ERCC1 *are among the few for which it has been shown that mRNA levels and repair activity correlate. The *ERCC1 *gene encodes a subunit of the nucleotide excision repair complex required for the incision step of NER [16,17]. Several factors indicate that the *ERCC1 *mRNA levels can be used as a proxy for DNA repair capacity. The mRNA levels of *ERCC1 *may correlate with DNA repair capacity in various tissues [11-13,15], and also predict the response to chemotherapy (high *ERCC1 *levels have been associated with tumor growth and lower levels have been associated with improved disease response) [15,18-21]. In addition, inter-individual variation of *ERCC1 *mRNA levels is much larger than the intra-individual variation [22], indicating that the mRNA level of *ERCC1 *may be used as a biomarker for NER.

The enzymes responsible for the BER process which repair damaged bases, abasic sites and single-strand breaks are initiated by specific DNA glycosylases. The DNA glycosylase OGG1 removes 8-oxo-guanine (8-oxoG) directly from damaged DNA [23,24]. Kondo et al concluded that there is a correlation between 8-OHdG levels and hOGG1 expression in CRC tissue [14]. Kondo et al also concluded that colorectal carcinoma cells but not adenoma cells are exposed to more oxidative stress than corresponding normal tissue [25]. Previous studies have shown that oxidative stress is generally proportional to *OGG1 *mRNA expression and activity [10,26,27]. To the best of our knowledge, it has not been investigated if increased *OGG1 *expression is an early or late event in the adenoma-carcinoma sequence. It is therefore of interest to study and compare the expression of *OGG1 *in adenoma and carcinoma tissues.

There are several responses to DNA damage including induction of apoptose to eliminate damaged cells. The putative apoptosis controlling gene *RAI *was included to determine if the expression of *RAI *was coordinated with the expression of *ERCC1*. Both genes are located on chromosome 19q13.2. The RelA associated inhibitor (*RAI*) gene, encodes an inhibitor of the RelA subunit of the transcription factor NF-κB [28]. The inclusion of this gene is more explorative but previous studies suggest that *RAI *is involved in carcinogenesis [29]. High levels of RAI seem to promote apoptosis while low levels of RAI may inhibit apoptosis, although the literature is not clear on this issue [28,30]. This is to the best of our knowledge the first study to investigate if increased *RAI *expression is an early or late event in the adenoma-carcinoma sequence.

In this study we have measured mRNA levels of *ERCC1, OGG1 *and *RAI *in patients with colorectal adenomas and carcinomas (normal colonic mucosa and lesions).

The main focus was to investigate if increased expression of DNA repair genes is an early or late event in the adenoma-carcinoma sequence. By studying repair genes in the various stages of the adenoma-carcinoma sequence, we may gain new information on the significance of repair capacity in normal and neoplastic tissues during transition.

## Methods

The KAM biobank (Kolorektal cancer, Arv og Miljø) is based on the screening group of the Norwegian Colorectal Cancer Prevention study (The NORCCAP study) in the county of Telemark [31]. The ID number for the NORCCAP study at clinicaltrials.gov is I NCT00119912 [32]. The KAM biobank has previously been described by Hansen et al. [33]. In the present study we have selected and analyzed cases with adenocarcinomas (9) and cases with adenomas (36). The sampling pool of cases contains tissue (normal colonic mucosa and lesion) from 180 CRC cases and 242 adenoma cases. The cases are matched for age and gender. The adenocarcinomas were collected prior to chemo- or radiotherapy treatment. The tumor histology of the adenomas was examined independently by two specialist histopathologists in order to determine the tumor stage as either mild/moderate (n = 21) or severe (n = 15) dysplasia (tumor cellularity 60–80%). They reached the same conclusion in all cases. All cases completed a questionnaire containing information on a family history of cancer and the included cases had no known personal history of cancer. The KAM cohort consists of an ethnic homogeneous group of Norwegian origin. The KAM study is approved by the Regional Ethics Committee and The Data Inspectorate.

Total RNA was purified from normal colonic mucosa and lesions as recommended by the manufacturers using NucleoSpin DNA II kit (Macherey Nagel). The tissue had been snap frozen in liquid N_2 _before RNA purification. RNA purification included a DNAse treatment. The cDNA synthesis was performed on ca. 200 ng RNA in a final volume of 10 μl using Gold RT-PCR kit (Applied Biosystems, Nærum, Denmark).

Quantitative PCR was performed on ABI 7500 sequence detection system (Applied Biosystems, Nærum, Denmark) in universal mastermix (Applied Biosystems, Nærum, Denmark) using 100 nM Taqman probe and 200 nM primers for *ERCC1 *and for *OGG1*, 100 nM probe and 600 nM primers for *RAI*. *ERCC1*, *OGG1 *and *RAI *primers and probes were obtained from TAG Copenhagen, Copenhagen, Denmark. The *OGG1 *probe recognizes all splice forms of *OGG1*. The expression was normalized to 18S rRNA. 18S primers and probes were commercially available from Applied Biosystems, Nærum, Denmark.

The quantification of mRNA levels has been described previously [22,34]. The probes and primers have been validated, and the PCR was shown to be quantitative over a range of 130-fold dilution for *ERCC1 *and *OGG1 *and over a 32-fold range for *RAI*. mRNA measurements were excluded if the 18S content was below the detection level of the individual mRNA defined by the validation experiments. *ERCC1*, *OGG1, RAI *mRNA and 18S levels were quantified in triplicate in separate wells. The average mRNA level of the specific mRNAs was then normalized to the average 18S level. The average standard deviation on triplicates was 15 %. The standard deviations on repeated measurements of the same sample (the control) in separate experiments were 34%, 21% and 25% for *ERCC1*, *OGG1 *and *RAI*, respectively, indicating the day-to-day variation of the assay. Independent PCR reactions of the same samples yielded a correlation coefficient of 0.97. Negative controls (where the RNA was not converted to cDNA) and positive controls were included in all sets.

For comparison of different mRNA expression levels between normal tissue and lesions in cases we used paired t-test. When comparing expression levels in normal colonic mucosa from all cases, and lesions in all the cases separately, a one-way ANOVA test was used. P values < 0.05 were considered significant for all statistical tests. For trend analysis of normal colonic mucosa in *OGG1 *and *RAI *we used logistic regression after dividing cases in mild/moderate dysplasia (1), severe dysplasia (2) and CRC (3). MiniTab Statistical Software, Release 13.1 Xtra (Minitab Inc.) was used for statistical calculations.

## Results

**Figure 1:**
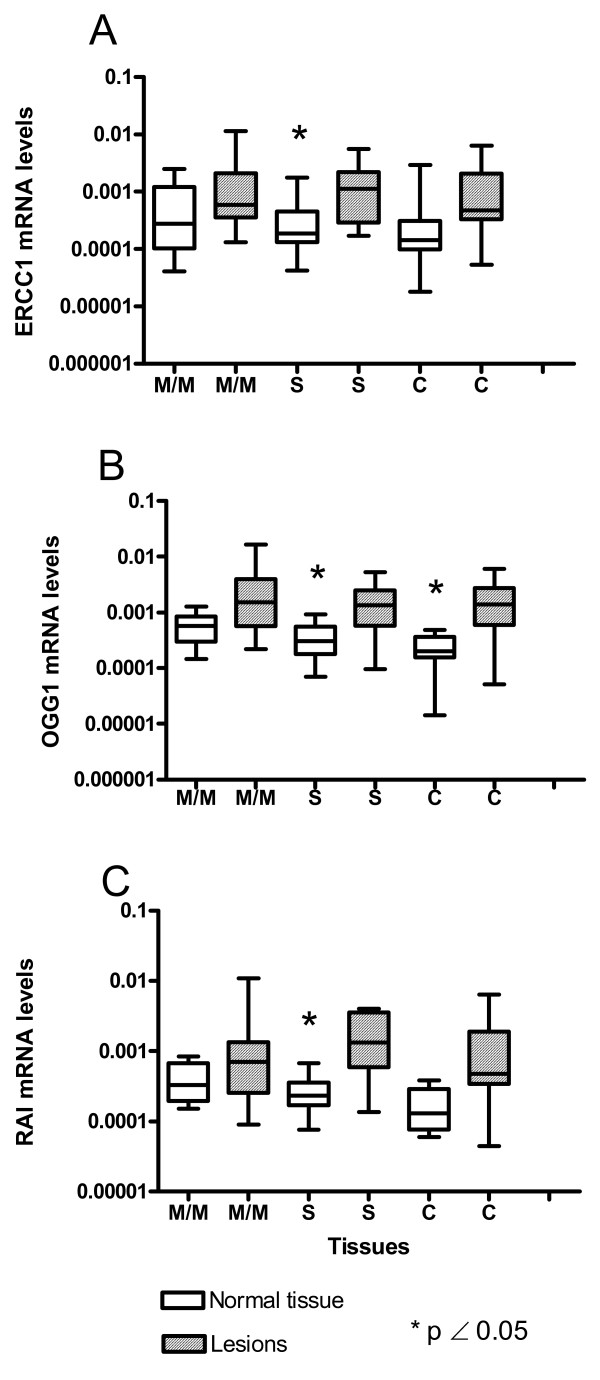
mRNA expression of *ERCC1*, *OGG1 *and *RAI *in lesions. mRNA levels were normalized to the 18S mRNA level. The classification of lesions are; M/M: adenoma with mild or moderate dysplasia; S: adenomas with severe dysplasia; C: adenocarcinomas. Open squares designate the expression level in normal colonic mucosa while crossbars designate the expression in lesions. A: *ERCC1*, B: *OGG1*, C: *RAI*. * designate statistically significant different mRNA levels in normal colonic mucosa compared with lesions in the same persons (p < 0.05).

The distributions of age and gender are described in Table 1. Cases are divided into three groups according to diagnosed degree of dysplasia; adenomas (mild/moderate or severe dysplasia) and carcinomas. mRNA expression in normal colonic mucosa and all lesions for the three genes, *ERCC1*, *OGG1 *and *RAI *are displayed separately in Figure 1 (A, B, C), and mean values are displayed in Table 2.

For all three genes, *ERCC1, OGG1 *and *RAI*, a comparison of expression levels between normal colonic mucosa and lesions in all cases combined (adenomas and CRC) yielded significantly higher expression levels in lesions, 3.3-fold P = 0.005, 5.6-fold P < 3*10^-5 ^and 7.7-fold P = 0.0005, respectively. When comparing expression levels between normal colonic mucosa and lesions separately for each case group (mild/moderate dysplasia, severe dysplasia and CRC), we detected significantly higher expression levels for *ERCC1 *and *RAI *in cases with severe degree of dysplasia and for *OGG1 *we detected significantly higher expression levels in cases with severe degrees of dysplasia and CRC (Table 2).

When comparing expression levels of *ERCC1 *in lesions only, we detected no difference between the adenoma and CRC case groups, P = 0.836. This was also true for normal tissue, P = 0.291, (Fig 1A).

The level of *OGG1 *expression when comparing lesions did not differ between adenoma and CRC cases P = 0.34, (Fig 1B), but for normal colonic mucosa the *OGG1 *expression in CRC patients was statistically significantly lower than in normal colonic mucosa from adenoma cases with mild/moderate dysplasia, P = 0.012, (Fig 1B). A trend of lowered mRNA expression with increasing degree of dysplasia was also observed in normal colonic mucosa, P = 0.003.

The level of *RAI *expression when comparing lesions did not differ between adenoma and CRC cases, P = 0.909, (Fig 1C). The level of expression in normal colonic mucosa was however significantly lower in CRC cases compared to adenoma cases with mild/moderate dysplasia, P = 0.043, (Fig 1C). A trend of lowered mRNA expression with increasing degree of dysplasia was also observed in normal colonic mucosa, P = 0.011.

## Discussion

In this study we have measured mRNA levels of *ERCC1, OGG1 *and *RAI *in patients with colorectal adenomas and carcinomas (normal colonic mucosa and lesions). We have compared expression levels between corresponding normal colonic mucosa and lesions in both adenoma and CRC cases. In order to see if an increase in expression is an early or late event in the adenoma-carcinoma sequence we have also compared expression levels between case groups in normal colonic mucosa and lesions separately.

*ERCC1 *expression was statistically significantly higher in lesions than in the corresponding normal tissue when all cases were combined. For *OGG1 *and *RAI*, a similar increase in mRNA levels in lesions compared to the corresponding normal colonic mucosa was detected. Previous studies have shown that the mRNA levels of *ERCC1 *[11-13,15,35] and *OGG1 *[10,14,27] correlate with the DNA-repair capacity in various tissues. The increased mRNA level detected in lesions in this study may therefore be interpreted as enhanced DNA repair activity.

Low expression of DNA repair genes in lymphocytes have previously been shown in patients with breast cancer, lung cancer and cancer of the head and neck [4-7]. This has been interpreted as low DNA repair gene expression is a risk factor for cancer. For both *OGG1 *and *RAI *we found that the expression was lower in normal colonic mucosa from CRC patients than in normal colonic mucosa from persons with mild/moderate dysplasia. This lowered mRNA level can either be interpreted as a risk factor for adenoma and CRC development but it can also be a consequence of the same.

When comparing different lesions in the adenoma-carcinoma sequence, we detected no significant difference in expression. This was true for all three genes, *ERCC1*, *OGG1 *and *RAI*. This may indicate that increased expression of DNA repair is an early event in the adenoma-carcinoma sequence. The result for *RAI *may be an indication that *RAI *and *ERCC1 *are coordinated as they both are located on chromosome 19q13.2. The NER gene *XPD *is also located on chromosome 19q13.2 and a previous study by Vogel et al concluded that *ERCC1 *and *XPD *mRNA quantities were highly correlated [13]. The *RAI *mRNA is many-fold induced during apoptosis and the induction is essential to apoptosis (Magdalena Laska et al, manuscript in preparation). Increased *RAI *expression has previously been observed in acute leukaemia cells [36]. We and others have found that *RAI *mRNA is also often increased in cancer cells (Bergamaschi et al [30] and this paper). We interpret this increase as an attempt on the part of the cancer cells to activate the apoptotic pathway. However, that attempt is presumably abortive as the cancer cells continue to multiply. Possibly, the well-known derangement of the intracellular control in cancer cells may have destroyed other essential factors and/or the downstream response.

Higher expression of DNA repair genes in lesions compared with normal colonic mucosa may indicate chemoresistance. The difference in expression levels between normal colonic mucosa and lesions for both *ERCC1 *and *OGG1 *could mean that lesions in general have a higher DNA repair capacity and therefore is less sensitive to DNA damaging agents such as chemotherapy. Shirota *et al *showed that CRC patients with low levels of intratumoral mRNA *ERCC1 *expression had significantly better survival than patients with high expression, when treated with 5-FU and oxaliplatin as second- or third-line chemotherapy [21].

Hereditary cancer predisposition syndromes have provided insight to the importance of DNA repair, but there are only limited data on inter-individual variation in DNA repair and susceptibility to sporadic cancer. Vogel *et al *detected an inter-individual mRNA variation for *ERCC1 *and *OGG1 *that was 5–10 fold in healthy volunteers [22], and a study by Paz-Elillzur *et al *concluded that low OGG1 activity was associated with an increased risk of lung cancer [37]. However, in the only prospective study of mRNA levels of DNA repair genes and *RAI*, low expressions of *ERCC1*, *XPD *or *RAI *were not associated with increased risk of lung cancer [38]. In a study by Hansen *et al *[39] the *OGG1 *Ser^326^Cys polymorphism, which has a reduced glycosylase activity [40], was not associated with an increased risk of colorectal adenomas or carcinomas.

## Conclusion

In conclusion, mRNA levels of *ERCC1 *and *OGG1 *were up-regulated in both colorectal adenomas and carcinomas compared to corresponding normal colonic mucosa, indicating that increased expression of defense genes is an early event in the progression of colorectal adenomas to CRC. There are some issues that must be considered when evaluating our findings. The number of cases is relatively small, therefore there is a possibility that the reported findings are due to chance. Larger prospective studies would be required to clarify this issue.

## Competing interests

The author(s) declare that they have no competing interests.

## Authors' contributions

MS prepared samples for analysis and contributed with the data analysis. She prepared the first draft of the paper.

CFS prepared samples for analysis and contributed with the data analysis. She also contributed to the manuscript.

ILH participated in collection and quality control of the questionnaires.

UBV, HW and BAN performed quantification of RAI and OGG1

EHK brought the idea of the KAM study and organized it.

All authors discussed the results, contributed to interpretation of the results and the final manuscript.

## Pre-publication history

The pre-publication history for this paper can be accessed here:



## Figures and Tables

**Table 1:** Distribution of age and gender.

	Cases		
	
	Adenomas^a^		CRC
	
	Mild/moderat dysplasia	Severe dysplasia	
	(n = 21)	(n = 15)	(n = 9)
Men^b^	16 (76%)	13 (87%)	7 (78%)
Women^b^	5 (24%)	2 (13%)	2 (22%)
Mean age +SD^c^	59.0 ± 3.1	58.4 ± 4.3	61.67 ± 9.1

a The adenomas are divided in groups according to diagnosed degree of dysplasia (mild/medium or severe).

b There is no significant difference in the number of males and females between the three case groups (χ^2 ^test).

c There is no significant difference in age between the three case groups (Mann-Whitney test).

**Table 2:** Mean normalized *ERCC1*, *OGG1 *and *RAI *mRNA levels in normal colonic mucosa and lesions.

	*ERCC1*				*OGG1*				*RAI*			
	Mean level × 1000^a^				Mean level × 1000^a^				Mean level × 1000^a^			
	
Type of tissue	Normal	Lesions	P^b^	95% CI	Normal	Lesions	P^b^	95% CI	Normal	Lesions	P^b^	95% CI
All cases combined	0.55	1.8	**0.005**	**(0.4 ;2.2)**	0.33	1.83	3*10^**-5**^	**(0.9 ; 2.1)**	0.26	2.0	**0.0005**	**(0.8 ; 2.7)**
												
Adenomas^c^												
Mild/moderate	0.76	2.2	0.151	(-0.9 ; 3.5)	0.44	2.13	0.069	(-0.2 ; 3.6)	0.31	3.1	0.135	(-1.6 ; 6.6)
Severe	0.39	1.5	**0.027**	**(0.15 ; 2.1)**	0.35	1.68	**0.004**	**(0.5 ; 2.2)**	0.28	1.85	**0.0014**	**(0.4 ; 1.5)**
												
CRC	0.16	1.5	0.109	(-0.4 ; 3.1)	0.24	1.83	**0.034**	**(0.2 ; 3.0)**	0.18	1.46	0.09	(-0.3 ; 2.8)

^a) ^Since *ERCC1*, *OGG1 *and *RAI *expression is expressed as a ratio, normalized, to 18S rRNA expression, it is unit-less.

^b) ^Comparison of the expression levels in lesions and normal colonic mucosa from the same persons.

^c) ^The adenomas are divided in groups according to mild/moderate dysplasia or severe dysplasia.
